# Time-Course Proteome Analysis Reveals the Dynamic Response of *Cryptococcus gattii* Cells to Fluconazole

**DOI:** 10.1371/journal.pone.0042835

**Published:** 2012-08-06

**Authors:** Hin Siong Chong, Leona Campbell, Matthew P. Padula, Cameron Hill, Elizabeth Harry, Simone S. Li, Marc R. Wilkins, Ben Herbert, Dee Carter

**Affiliations:** 1 School of Molecular Bioscience, University of Sydney, Sydney, New South Wales, Australia; 2 ithree institute, University of Technology, Broadway, New South Wales, Australia; 3 School of Chemistry and Biomolecular Sciences, Macquarie University, North Ryde, New South Wales, Australia; 4 School of Biotechnology and Biomolecular Sciences, University of New South Wales, Kensington, New South Wales, Australia; Research Institute for Children and the Louisiana State University Health Sciences Center, United States of America

## Abstract

*Cryptococcus gattii* is an encapsulated fungus capable of causing fatal disease in immunocompetent humans and animals. As current antifungal therapies are few and limited in efficacy, and resistance is an emerging issue, the development of new treatment strategies is urgently required. The current study undertook a time-course analysis of the proteome of *C. gattii* during treatment with fluconazole (FLC), which is used widely in prophylactic and maintenance therapies. The aims were to analyze the overall cellular response to FLC, and to find fungal proteins involved in this response that might be useful targets in therapies that augment the antifungal activity of FLC. During FLC treatment, an increase in stress response, ATP synthesis and mitochondrial respiratory chain proteins, and a decrease in most ribosomal proteins was observed, suggesting that ATP-dependent efflux pumps had been initiated for survival and that the maintenance of ribosome synthesis was differentially expressed. Two proteins involved in fungal specific pathways were responsive to FLC. An integrative network analysis revealed co-ordinated processes involved in drug response, and highlighted hubs in the network representing essential proteins that are required for cell viability. This work demonstrates the dynamic cellular response of a typical susceptible isolate of *C. gattii* to FLC, and identified a number of proteins and pathways that could be targeted to augment the activity of FLC.

## Introduction

Fungal diseases are an emerging problem worldwide. Globally, immunosuppression due to HIV/AIDS is the most important risk factor for developing systemic fungal infections. In the developed world, medical advances such as organ transplants and chemotherapy, along with increases in diabetes, obesity and old age, cause vulnerability to a wide spectrum of fungal infections. Ongoing changes in fungal ecology and range expansion have also been implicated in some recent outbreaks of previously uncommon mycoses [1], [2], [3].

Aggressive antifungal treatment is frequently required to resolve serious mycoses, especially in debilitated hosts. Unfortunately, the available antifungal therapies are very limited in scope and spectrum. Current antifungal drugs work via only five different mechanisms, and of these only three classes of drugs can be used to treat systemic infections effectively [4]. The polyene class includes amphotericin B, which is fungicidal and highly effective against most fungal pathogens but also toxic to mammalian cells and is associated with serious side effects. Azole-based drugs, which include fluconazole and newer triazoles, are generally well tolerated but cannot be used during pregnancy, are ineffective against some important pathogens including *Fusarium, Scedosporium* and the zygomycetes, and can induce resistance. Echinocandins are the newest class of antifungal drug and are highly effective against *Candida* and *Aspergillus* species but show no activity against *Cryptococcus*, *Fusarium* and the zygomycetes, and resistance is an emerging issue [5].

The development of new antifungals is difficult as targeted structures or cellular pathways must be sufficiently conserved among the fungi to ensure broad-spectrum coverage (and hence a profitable market for pharmaceutical companies), but sufficiently divergent between fungi and animals to prevent cross-reactivity and undesirable side effects. A promising alternative approach to the *de novo* development of new drugs is to develop therapies that work in synergy with existing drugs, either by targeting cellular pathways that enhance drug action or block the development of resistance, or by augmenting the host response, thereby speeding up clearance of the pathogen.

Cryptococcosis is one of the most important systemic mycoses, and *Cryptococcus* has become an important model organism for studies of fungal virulence and host response. *Cryptococcus neoformans*, a major AIDS-related pathogen, causes over one million infections and more than 650,000 deaths per year, rivaling tuberculosis in importance in many parts of the world [6]. The related species *C. gattii*, although rarely found in the immunocompromised, is an important primary pathogen and is responsible for a significant ongoing outbreak in otherwise healthy people and animals in the Pacific Northwest of Canada and the United States [1]. Most *Cryptococcus* strains are susceptible to FLC, which is widely used in prophylactic and maintenance therapy. However, some *C. gattii* isolates have reduced FLC susceptibility, despite having never been exposed to azole-based drugs. This appears to segregate with genotype, such that genotype VGII is significantly less susceptible than genotypes VGI and VGIII [7], [8]. The reason for these different responses is not known.

Understanding how a pathogen responds to a drug is today best achieved using systems biology approaches that enable the entire cellular response to be investigated. Proteomics, where expressed proteins are identified and quantified, has been employed to investigate the response of *Candida albicans* and *Candida glabrata* to antifungals such as azoles and polyenes [9], [10]. Few proteomic studies have been conducted on *Cryptococcus*, and none have investigated its response to antifungals. The ability to profile the global response to an antifungal agent makes it possible to identify proteins that are not directly affected by the antifungal drug but are modulated in response to the stress that the drug imposes. Since these may be important for allowing the fungal cell to survive they are potential targets for use in synergistic therapies. In this study, we analyzed the proteome of a representative, moderately susceptible strain of *C. gattii* VGII during growth in the presence and absence of FLC. The aims of this work were 1) to determine a baseline proteome for *C. gattii* during normal early to mid log growth; 2) to characterize proteins that are differentially expressed during FLC treatment; and 3) to use network analysis to identify key cellular products or pathways that might form suitable targets for synergistic therapies with FLC. Cellular proteins involved in stress response, macromolecule biosynthesis and energy production for growth were affected during growth and in response to FLC, and the changes in expression of these proteins over time were dynamic and multiple. Network analysis of these differentially expressed proteins revealed co-ordinated processes involved in the drug response that may be suitable targets for synergistic therapies with FLC.

## Results

### Cell size and protein yield increase with FLC treatment

**Figure 1:**
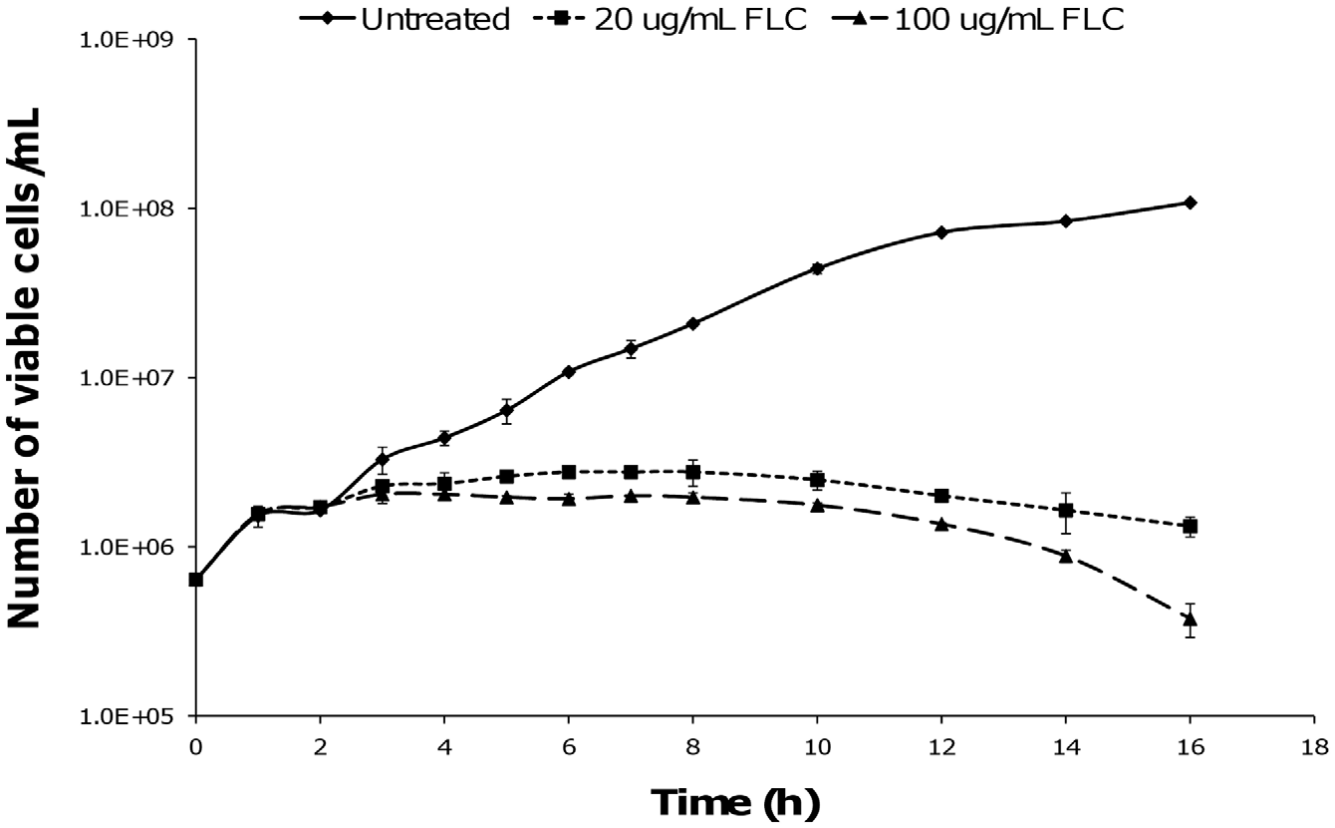
The effect of FLC on growth of *C. gattii* VGII in YPD media. A susceptible strain V5 was tested in the presence of FLC at two different concentrations. Growth of V5 was inhibited after 2 h of drug exposure. Data are shown as mean +/− standard error.

**Figure 2:**
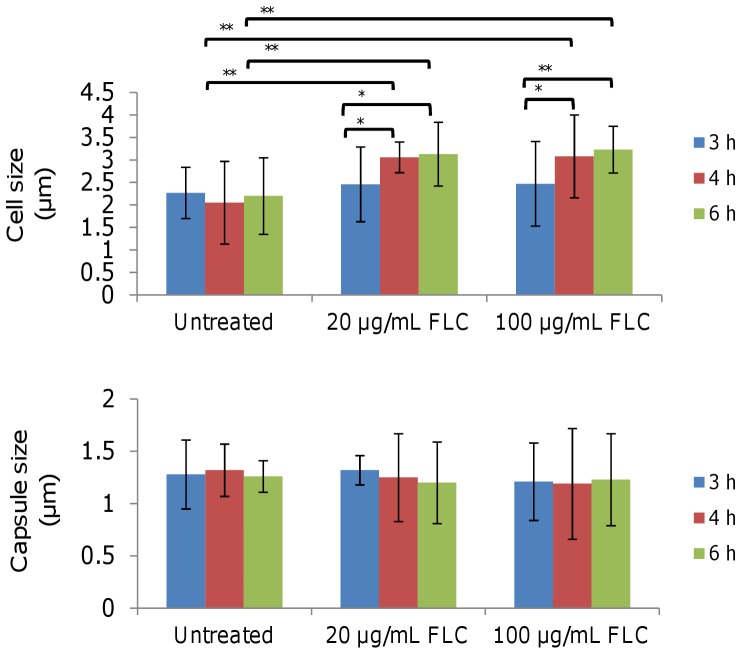
The effect of FLC treatment on the morphology of *C. gattii* cells. Data are shown as mean +/− standard error. * indicates P<0.05 and ** indicates P<0.001.

Growth of a representative, moderately susceptible *C. gattii* isolate was initially assessed in the presence of 20 and 100 µg/mL FLC. Growth of all cell cultures was affected after exposure to FLC for 2 hours (Figure 1), and cell imaging revealed changes to cellular morphology during FLC treatment (Figure 2). There were no significant differences in capsule size among cells treated with or without FLC at each time point (*p*≥0.65). However, cells treated with either concentration of FLC for 4 h and 6 h were significantly larger than the untreated cells (*p*<0.001) and the cells treated with FLC for 3 h (*p*≤0.004). Cells treated with 20 µg/mL of FLC had a similar size to cells treated with 100 µg/mL of FLC at three different time points (*p*≥0.25). There was no difference in the size of cells treated with FLC for 4 h and 6 h (*p*≥0.15), or between untreated cells and those treated with FLC for 3 h (*p*≥0.07).

Total protein in samples was quantified to assess the yield of protein per *C. gattii* cell volume. Protein yield from the untreated cells was similar across the three different time points (∼3.6×10^−7^ µg/µm^3^; *p*≥0.074). In contrast, the yield from the samples treated with FLC dropped significantly from ∼3.9×10^−7^ µg/µm^3^ at 3 hours to ∼2.6×10^−7^ µg/µm^3^ and ∼3.1×10^−7^ µg/µm^3^, for 4 and 6 h respectively (*p*≤0.03). These were also lower than the respective untreated samples at 4 and 6 h (*p*<0.012) but higher than the untreated samples at 3 h (*p* = 0.027).

### Numerous cellular responses occur during growth in the presence or absence of FLC

Changes in protein expression were analysed to assess differential protein expression patterns during growth of *C. gattii* in the absence or presence of FLC (Table S1). The differentially regulated proteins were grouped into ten functional groups: stress response, signal transduction, ribosomal proteins, sugar or lipid metabolism, protein or amino acid metabolism, plasma membrane proteins, nuclear proteins, cytoskeleton proteins, miscellaneous proteins and uncharacterized proteins with unknown functions.

**Figure 3:**
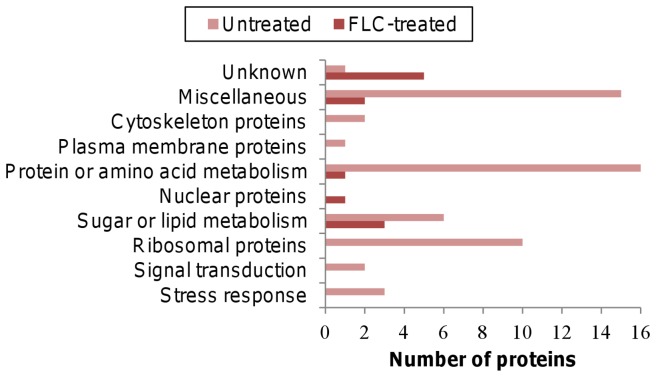
Categories of proteins that were present only in the FLC-treated or in the untreated samples. Proteins involved in energy production, protein metabolism and ribosomal protein synthesis were substantially reduced in the FLC-treated cells, while the number of unknown proteins increased in the FLC-treated cells.

In the untreated samples, a total of 195 proteins were identified, while 152 proteins were identified across all of the FLC-treated samples. When all of the proteins identified were compared, 12 were found only in the FLC-treated samples. The majority of proteins found only in the untreated samples had functions involving protein or amino acid metabolism, ribosomal biosynthesis or energy metabolism including respiration and glycolysis. In contrast, most of the proteins found only in the FLC-treated samples were uncharacterized proteins without known functions, or were involved in protein and sugar metabolism (Figure 3; Table S1).

Protein expression profiles of the untreated and FLC-treated samples at the middle (4 h) and late (6 h) stages of drug response were compared to those at the early (3 h) stage of the drug response to examine how protein expression patterns changed over time (Table S1). During normal (drug-free) growth, 99 proteins were induced (ie. only found in middle-late stage) or differentially expressed. Of these, 41 proteins were present only in the 6-hour sample, with the majority having a role in protein or amino acid metabolism or energy production (respiration, glycolysis and tricarboxylic acid cycle), and the remainder involved in the stress response and in the biosynthesis of ribosomes, the plasma membrane and the cytoskeleton. These results are consistent with normal early exponential phase growth, as the nutrients were sufficient for cells to multiply without any major stress, and in doing so the cells produced more proteins. During growth in the presence of FLC, 56 proteins were induced or differentially regulated in mid-late growth (Table S1), with 30 of these found exclusively in the 6-h FLC-treated samples. Compared to the change in expression patterns observed during normal growth, there were reduced numbers of various proteins involved in energy production and protein synthesis, particularly ribosomal proteins, consistent with the slowing of active metabolism and growth.

### Dynamic overview of the antifungal response to FLC over time

To investigate the metabolic pathways affected by FLC, protein expression profiles were compared between the FLC-treated and the untreated cells at each of the three time points (Table S2). In the response to FLC over time, reduced levels of ribosomal proteins and increasing levels of heat shock proteins, plasma membrane proteins, and various proteins involved in sugar metabolism, ATP synthesis and the mitochondrial respiratory chain were observed. Five proteins were consistently up-regulated or induced by FLC, and these included 14-3-3 protein, 40S ribosomal protein S7, 60S ribosomal protein L6, cytochrome c oxidase subunit 2 and peripheral-type benzodiazepine receptor. Two proteins involved in fungal-specific pathways were differentially expressed in response to FLC. Of these, G protein beta subunit Gib2 was down-regulated at each time point, while isoprenoid biosynthesis-related protein was up-regulated at 6 hours.

### Co-ordinated processes involved in drug response revealed by network analysis

**Figure 4:**
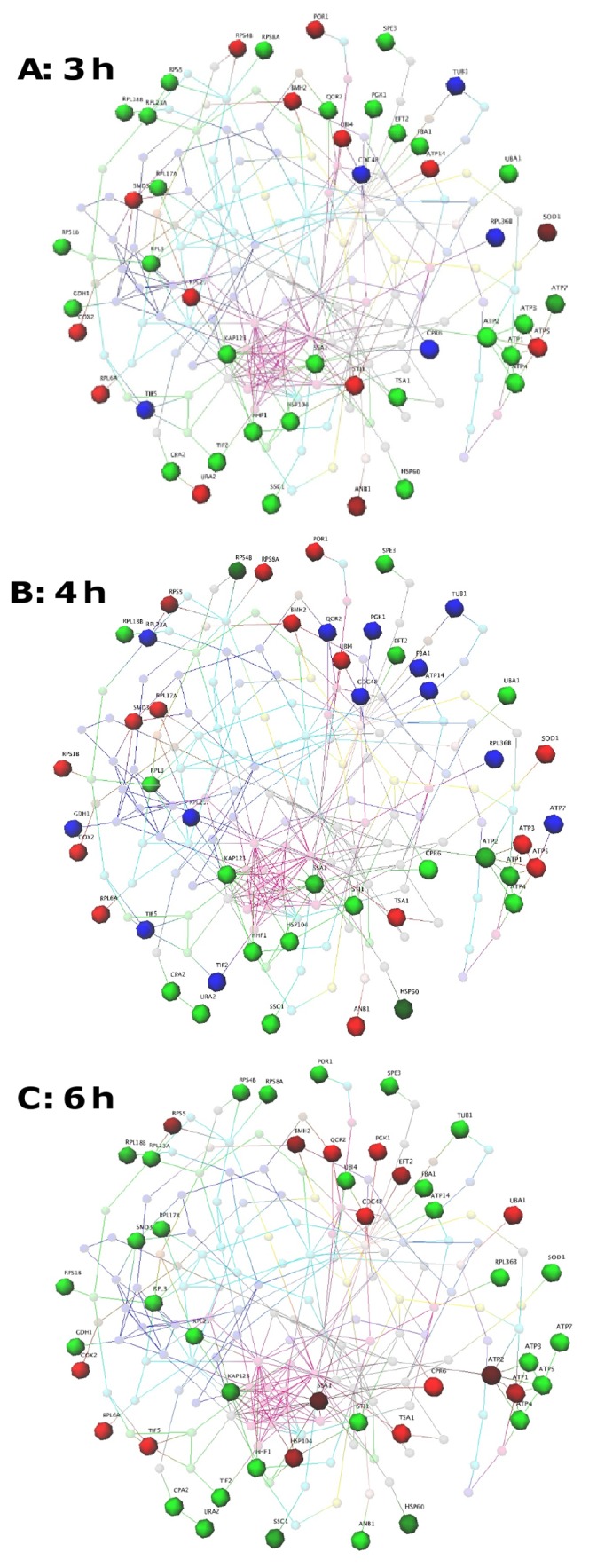
Drug response network analysis based on the change in protein expression over time. Spheres represent proteins and lines connecting spheres indicate interactions between proteins. Large spheres are proteins differentially expressed in response to FLC; red indicates proteins induced or up-regulated; green indicates proteins suppressed or down-regulated; and blue indicates no data at that time point. Smaller spheres are colour-mapped according to Gene Ontology biological process. The network analysis reveals correlated processes in response to FLC treatment from 3 h to 6 h time points. See Figure 5 for the Gene Ontology terms of these proteins. [GEOMI Force Directed Layout: spring = 50, origin = 80, repulsion = 12, planar = 100].

**Figure 5:**
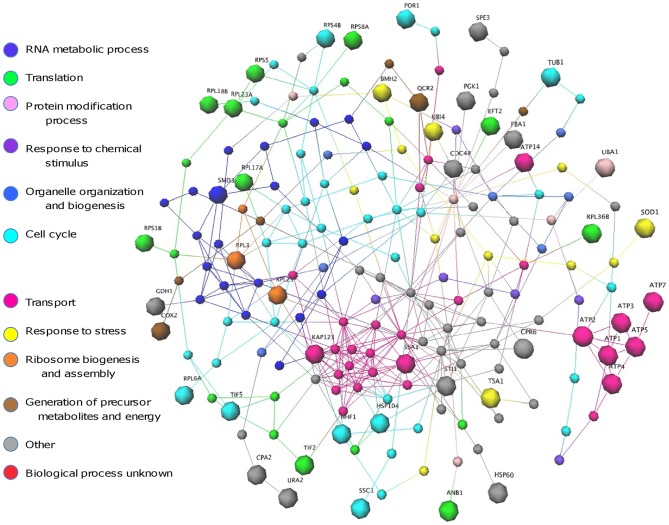
Drug response network drawn as in **Figure 4** and coloured using Gene Ontology data. Network analysis reveals clustering of protein with similar functions and hub proteins with a high degree of connectivity. Potential targets for synergistic therapies are indicated in Figure S2. [GEOMI Force Directed Layout: spring = 50, origin = 80, repulsion = 12, planar = 100].

As the proteome analysis revealed dynamic changes in protein expression in cells treated with FLC over time, network analysis was used to gain further insight into how the differentially regulated proteins might interact with each other, and how the cells co-ordinate their response to FLC over time. High-quality protein-protein interaction data for the yeast *Saccharomyces cerevisiae* were used to generate the networks, as protein-protein interactions have not yet been studied on a genome-wide scale in *Cryptococcus*. Corresponding yeast homologs were identified for each of the differentially expressed proteins listed in Table S2 (Table S3). Seventy-seven of the 82 cryptococcal proteins had a yeast homolog with an E value of less than 10^−9^. Of these, 47 were present in the yeast protein-protein interaction datasets used, which only include interactions that have been experimentally validated [11], [12]. Using these 47 yeast proteins, two different types of networks were built to investigate the overall cellular drug response in *C. gattii*. These were seeded with the 47 homologs and extended to show the most direct connections (shortest path) between these homologs using known protein-protein interactions. Figure 4 depicts expression networks for the three time points (3, 4 and 6 h) and shows how the expression of proteins differentially regulated by FLC treatment changes over time (see Figure S1 for larger version of these networks). Figure 5 shows the same network with Gene Ontology (GO) data mapped to the differentially expressed proteins (large spheres), the interacting proteins (small spheres) and the interactions (lines) that join them.

A total of 182 yeast proteins, including the 47 homologs of the differentially expressed *C. gattii* proteins, and 392 interactions, were present in the networks. Among the differentially expressed *C. gattii* proteins mapped to the network, three warrant further investigation as putative drug targets due to their significance in yeast cells and their interactions with multiple partners (Figure 5; also see Figure S2C): ATPase involved in ubiquitin-mediated protein degradation (yeast homolog: CDC48); ATPase belonging to heat shock protein 70 family (yeast homolog: SSC1); and core Sm protein Sm D3 of the Sm ring complex required for the biogenesis of the small nuclear ribonucleoproteins (yeast homolog: SMD3). Ten interacting proteins (thirteen essential proteins in total, including the three mentioned above) that were not directly responsive to FLC but are known to be essential in yeast were also identified (Figure S2C): karyopherin α homolog (SRP1); two karyopherin β subunits (PSE1 and KAP95); the catalytic subunit of the main cell cycle-dependent kinase (CDC28); ubiquitin-like protein (SMT3); and five subunits of the nuclear pore complex (NUP1, NUP49, NUP57, NUP116, and NUP145).

Proteins with the same colour, indicating that they shared GO term and assigned function, tended to cluster together. For example, seven ATP synthesis proteins, including six that were differentially expressed (ATP1-5 and ATP7) and one interacting protein (ATP6), formed a tightly interacting group (Figure S2B; circled in red). All of these proteins belong to components of mitochondrial F1F0-ATP synthase, which is responsible for producing ATP required to power and sustain virtually all cellular processes in an organism. A group of proteins belonging to the nuclear pore complex, including the five essential proteins mentioned above, were highly interacting with each other (Figure S2C; circled in black). Similarly, coordinated changes in expression are seen between proteins that share similar activities (Figures 4 and 5). URA2 (bifunctional carbamoylphosphate synthetase-aspartate transcarbamylase) and its connecting partner CPA2 (large subunit of carbamoyl phosphate synthetase) both share carbamoyl phosphate synthase activity, and the change in STI1 (heat shock protein 90 co-chaperone) appears linked to changes in the expression of its interaction partners SSA1 and HSP104 (both heat shock proteins), such that these were both consistently co-expressed.

## Discussion

Antimicrobial treatment results in changes in both target and non-target cellular processes, with the latter generally evoked in response to slowed growth and stress as the cell attempts to cope with the damage induced by the antibiotic. The aim of this work was to analyze the overall cellular response in the soluble proteome of a typically sensitive *C. gattii* cell. Network analysis identified a number of key hub proteins in this response that govern a number of interacting cellular pathways; these warrant further investigation as putative targets to use synergistically with FLC.

The time-course study of differentially expressed proteins (Table S2), together with the network analysis (Figure 4), show clearly that *C. gattii* cells respond to FLC in a dynamic manner that operates in an integrated fashion, and that although replication and growth are impaired by FLC, many proteins are up-regulated, even after 6 hours of treatment. Particularly interesting in this regard is the clustering and coordinated regulation over time of proteins ATP1-7, which are components of mitochondrial F1F0-ATP synthase (Figures 4 and 5; also see Figure S2B). ATP synthase consists of a membrane-bound F0 component and a water soluble F1 component [13]. ATP1 and ATP2 (the α and β subunits) belong to the F1 component with ATP3 (γ subunit) forming the central stalk. ATP4-7 are part of the F0 complex, while ATP6 is a key component of the proton channel [14], [15]. In this study, induced expression of ATP5, which is essential for binding F1 and F0 and forming an active complex, was up-regulated at 3 and 4 hours, followed by up-regulation of ATP3 at 4 hours, then up-regulation of the core subunits ATP1 and ATP2 at 6 hours. This indicates a coordinated initiation of ATP synthase in response to FLC, which may be required to drive ATP-dependent efflux pumps. This observation of the systematic regulation of components of ATPase complex over time is consistent with their structure and biological function, and as well as aiding our understanding of the cellular response to FLC is a useful validation of the proteome data.

In addition to the ATP synthase complex and several mitochondrial respiratory proteins (cytochrome c oxidase subunit 2, inorganic phosphate transporter, NADH dehydrogenase and ubiquinol-cytochrome C reductase complex core protein 2), FLC treatment induced a number of other proteins involved in energy metabolism. Enzymes involved in the gluconeogenesis pathway, where pyruvate is converted back to glucose for energy production, were increasingly up-regulated over the experimental time-course, including enolase, fructose-bisphosphate aldolase, glyceraldehyde-3-phosphate dehydrogenase and phosphoglycerate kinase (Table S3). This suggests that a high level of energy was required for survival in response to FLC. One possible survival mechanism is to use an efflux system to remove toxic sterol intermediates or foreign compounds such as FLC. The efflux system is a survival and regulatory mechanism in yeasts that has been found to contribute to antifungal drug resistance [16], [17], and is an energy-dependent system driven by ATP. Nutrient stress during infection is likely to limit the ability of *C. gattii* cells to obtain all their energy needs, and this may aid in the ability of FLC, which is a fungistatic drug, to effectively contain and kill fungal cells.

FLC induced a number of proteins involved in glycolysis and carbohydrate metabolism, however proteins involved in the TCA cycle were not detected (Table S1), indicating that normal carbohydrate metabolism was no longer being achieved. This suggests that alternative pathways have been employed to use the products of the glycolytic pathway for growth and survival. The trehalose cycle is a pathway branched from glycolysis that yeast cells use to produce trehalose, a crucial protector of proteins and membranes against osmotic, heat shock and oxidative stress, starvation and toxicants [18], [19], and phosphoglucomutase catalyses the first reaction in this cycle. This enzyme was induced at the 6-h time point, suggesting that the trehalose cycle had been initiated to protect cells from FLC. This is further supported by an increased expression of UDP-glucose dehydrogenase, which produces UDP-glucose required for the trehalose cycle. Pyruvate kinase converts phosphoenolpyruvate to pyruvate, which is then catalyzed to produce acetyl-CoA in the glycolytic pathway. Acetyl-CoA can then enter the TCA cycle to generate ATP or can be used via the fatty acid biosynthesis pathway to produce sterols and lipids that are required for rigidity and integrity of the cell membrane [20], [21]. The induction of pyruvate kinase, fatty acid synthase complex protein and isoprenoid biosynthesis-related protein for sterol synthesis during growth in the presence of FLC suggests that the fatty acid biosynthesis pathway was the preferred pathway followed by glycolysis, and that cells might have been restoring membrane defects caused by FLC via this pathway. This is consistent with findings in *C. neoformans* [22] where transcriptional analysis suggested that fungal cells use lipid biosynthesis and metabolism to counterbalance ergosterol alteration in response to FLC.

Not surprisingly, FLC treatment induced the expression of a number of stress-related proteins. FLC is known to target ergosterol biosynthesis by inhibiting the activity of lanosterol demethylase [23], resulting in an increase in the accumulation of toxic intermediate lanosterol. In addition to toxic stress, FLC is likely to have induced membrane stress as cells grew larger but failed to divide (Figure 2). When the growth of plasma membrane is inhibited by FLC, continued synthesis of new proteins will eventually stretch the cells, leading to a stress response at the membrane. In this study, proteins found to function in protein synthesis (ATP-dependent RNA helicase Eif4a, elongation factor 1-gamma, eukaryotic translation initiation factor 5C homolog, MMS2, peptidyl-prolyl cis-trans isomerase D, translation elongation factor 2, and ubiquitin activating enzyme) were increasingly expressed during FLC treatment over time (Tables S2 and S3), indicating that new proteins were being synthesized. The present study also revealed that cells treated with FLC for over 4 h were significantly larger than those without FLC treatment (Figure 2). These findings suggest that the properties and functions of the cell wall and membrane were altered by the drug, as expected by its mechanism of action, resulting in the failure of proper cell growth and division. Previous studies of the effect of azoles on the morphology of *C. neoformans* have reported similar changes [24], [25]. The increase in protein synthesis, accompanied by impairment of cell division leading to enlarged cells, is likely to stress the cell membrane and cause an increase in various stress response proteins. FLC is also known to cause oxidative damage [10], and a consistent up-regulation of thiol-specific antioxidant protein 1 supports this view. These results are consistent with transcriptional profiling of *C. neoformans* in response to FLC, where an up-regulation of genes encoding proteins involved in the oxidative stress response including thiol-specific antioxidant protein 3 was also seen [22].

Although protein synthesis continued, the production of the majority of ribosomal proteins was down-regulated in response to FLC (Table S2), consistent with reduced protein yield for samples treated with FLC for 4 and 6 h, and with other studies of fungi treated with antifungal agents such as amphotericin B and FLC [26], [27]. However, two ribosomal proteins were consistently up-regulated: 40S ribosomal protein S7 and 60S ribosomal protein L6. 40S ribosomal protein S7 is crucial for cell viability in *S. cerevisiae* [28], and the 60S ribosomal protein L6 is an N-terminally acetylated protein component of the large ribosomal subunit. It is difficult to explain why certain ribosomal components would be up-regulated while overall the ribosomes are down-regulated, but this may point to functions that are independent of their roles in the ribosomes. Their consistent up-regulation suggests they might play an important role in the response to FLC and may help *C. gattii* maintain growth and survival.

Two proteins specific to fungal processes were differentially expressed during FLC treatment (Table S2). The first, G-protein beta subunit Gib2, was consistently down-regulated in response to FLC over time. This protein functions in a G-protein signaling system [29] and is essential for survival and viability in *C. neoformans*. However, although it is a Gβ-like/RACK1 protein homolog, Gib2 is an atypical protein that appears to be specific to *Cryptococcus*, which may limit its use as a broad-spectrum antifungal target. The second protein, isoprenoid biosynthesis-related protein, was not detected at 3 hours but became up-regulated at the later stage of the drug response. This protein is similar to yeast farnesyl pyrophosphate (FPP) synthetase, and is involved in sterol biosynthesis by producing isoprenes that are required at the early steps of ergosterol synthesis [30].

Two other proteins found consistently up-regulated during FLC treatment over time were 14-3-3 protein and peripheral-type benzodiazepine receptor (Table S2). 14-3-3 protein is highly conserved among the fungi and is involved in signal transduction, controlling many cellular processes such as pseudohyphal induction, bud cell development and virulence-associated morphogenesis [31], [32]. In *S. cerevisiae*, mutations in the 14-3-3 protein have been shown to confer resistance to rapamycin, an immunosuppressant drug [33]. In *Cryptococcus*, 14-3-3 protein is immunodominant in koalas with a subclinical *Cryptococcus* infection and in mice with pulmonary disease, and is up-regulated in rabbits with cryptococcal meningitis [34], [35]. These findings, and the consistent response of the 14-3-3 protein to FLC, indicate that it may play an important role in cell survival in the presence of drug and in the infected host. Peripheral-type benzodiazepine receptor, also known as translocator protein, is localized primarily on the outer mitochondrial membrane. It interacts with and utilizes porphyrins as endogenous ligands [36], and functions in the regulation of cholesterol transport for steroid biosynthesis in mammalian cells [37]. This protein is essential for stress adaptation in plants [38], thus the consistent up-regulation of this protein in *C. gattii* cells exposed to FLC may also be a stress response.

Not all proteins known to be involved in drug responses were detected in this analysis, and some of the most notable absences included lanosterol 14α-demethylase, and membrane transport proteins, such as ABC transporters and major facilitator superfamily transporters. Consistent with this, these proteins were not identified in a similar study that examined the soluble protein expression of *C. albicans* in response to ketoconazole [10]. However, expression of genes encoding these proteins has been seen in transcriptomic studies in *C. neoformans*, *S. cerevisiae* and *C. albicans* [22], [39], [40]. This indicates the limitations of gel-based proteomics, where membrane-bound and low abundance proteins are difficult to detect [41], [42] and argues for the use of complementary transcriptome and proteomic analyses, where possible.

The integrative network analysis identified a number of hub proteins that had multiple interaction partners and are likely to be essential for cell survival [43], [44], [45]. A number of other essential proteins were found based on information in *Saccharomyces* Genome Database and other published studies (Figure S2C; black arrows). These essential proteins are not fungal specific and play important roles in mammalian cells, and therefore cannot be the direct target of antifungal drugs. However, studies of drug synergy find the amount of drug needed to completely inhibit the growth of fungal cells can be reduced by more than 30-fold when used in combination [46], [47], [48], and these may be useful targets for combination therapies. As an example of this, inhibitors of calcineurin, which is important in both fungi and mammals, have been proposed for use in combination with FLC as in very small amounts these convert FLC from being fungistatic to being potently fungicidal [46].

Three of the thirteen essential proteins were differentially expressed in response to FLC. CDC48, which was highly up-regulated at 6 hours, is an ATPase involved in ubiquitin-mediated protein degradation, with an important role for maintaining the cell wall integrity during heat stress [49]. Depletion of CDC48 can trigger apoptosis [50], and cells are sensitive to oxidative stress [51]. SSC1, which was consistently down-regulated, is an ATPase belonging to heat shock protein 70 family that is a component of mitochondrial inner membrane [52]. This protein has a role in protein folding and translocation by using energy derived from ATP hydrolysis [53], [54]. Cells lacking functional SSC1 are not viable [55], and reduction of SSC1 function causes aggregation of mitochondria [56] and reduced fitness [57]. Finally, SMD3, which went from being up-regulated at 3 and 4 hours to down-regulated at 6 hours, belongs to the highly conserved Sm protein family and is associated with the biogenesis of the small nuclear ribonucleoproteins that function in post-translational modification of RNA [58], [59]. Cells with reduced expression of SMD3 show decreased levels of small nuclear ribonucleoproteins [58], leading to incomplete RNA processing. The other ten essential proteins found in the network include SRP1, PSE1, KAP95, CDC28, SMT3, NUP1, NUP49, NUP57, NUP116, and NUP145. SRP1, PSE1 and KAP95 are a group of karyopherins that interact with the nuclear pore complex and play a role in the transport of nuclear proteins and the regulation of protein degradation and desumoylation [60], [61], [62]. Disruption of these results in major defects in cell morphology and growth [61], [62], [63]. CDC28 is a catalytic subunit of the main cell cycle-dependent kinase that regulates cell growth by controlling the timing of mitotic commitment, bud initiation, DNA replication, spindle formation, and chromosome separation [64]; mutations result in defects in actin polarization and distribution with a failure to bud [65]. SMT3, a ubiquitin-like protein that regulates chromatid cohesion, chromosome segregation, proteolysis, DNA replication and septin ring dynamics [66], [67], [68], when mutated results in improper chromosome segregation [68], leading to a larger cell size as observed in this study. Finally, NUP1, NUP49, NUP57, NUP116, and NUP145 are a group of essential proteins belonging to the nuclear pore complex that interacts with SRP1, PSE1 and KAP95. Mutations in genes encoding these proteins cause defects in nuclear protein import, nuclear RNA export, nuclear pore complex assembly, and nuclear envelope integrity [69], [70], [71]. As all these essential proteins are involved in the overall control of a range of important cellular functions required to maintain the fitness of an organism, and although not fungal-specific, they may be useful starting points for identifying novel drug targets. Our future studies will assess whether depleting these proteins significantly affects the response of *C. gattii* and other fungi to FLC.

### Conclusion

This study examined the effect of FLC on the growth dynamics and the proteome of *C. gattii* using a label-free quantitative proteomic approach, and provided a broad overview of the response in this fungus to FLC. The analysis revealed numerous cellular proteins affected during growth and in response to FLC, and showed the dynamic changes in the expression of these proteins over time. The expression of proteins involved in macromolecule biosynthesis and energy production for growth was disrupted in the FLC-treated samples, and the abundance of proteins responsible for the stress response, energy production and macromolecule biosynthesis was altered throughout the three stages of drug exposure. An integrative network analysis revealed coordinated processes involved in the drug response and highlighted potential drug targets that may be suitable for synergistic therapies with FLC. Further studies are required to test if these can enhance antifungal activity and limit the development of drug resistance.

## Materials and Methods

### Strains and growth conditions

*Cryptococcus gattii* VGII strain V5, previously found to be moderately susceptible to FLC (MIC = 8 µg/mL) [7], was assessed for growth in the presence and absence of FLC (Sigma-Aldrich, Castle Hill, New South Wales) [7]. To establish growth curves, individual colonies were picked from one-day old agar plates and suspended in 25 mL of YPD broth (1% yeast extract, 2% peptone and 2% dextrose). Cultures were grown in a Ratek Orbital Mixer Incubator (Ratek Instruments Pty. Ltd., Victoria, Australia) at 37°C, 200 rpm to mid-logarithmic phase. When a cell density of approximately 8×10^5^ viable cells/mL was obtained, FLC was added to give a final concentration of 0, 20 and 100 µg/mL. Cultures were grown at 37°C, 200 rpm, and aliquots were removed at the indicated time points to assess cell numbers using a haemocytometer, and cell viability, which was determined by trypan blue staining (Sigma-Aldrich, Castle Hill, New South Wales). Three replicates were measured for each sample at each time point and averaged.

Based on the initial growth curves, 20 µg/mL of FLC was chosen as the most suitable concentration for proteome analysis. To ensure that any changes to the proteome were most likely to be a result of drug exposure, and not due to secondary effects (ie. nutrient starvation or cell death), V5 cells were initially harvested for protein extraction at 3 h, when they were beginning to respond to the presence of FLC but there was only a small difference in growth of the treated and untreated cells. Subsequent time points of 4 and 6 h were selected, with the 4 h time point showing growth in the presence of FLC had started to slow; and 6 h where a clear difference between the treated and untreated cultures was seen.

### Cell imaging

Aliquots of V5 cultures removed at 3, 4 and 6 h were visualised using a Leica DME microscope (Leica Microsystems, Wetzlar, Germany) equipped with an OMCAM30 3.0 megapixel USB eyepiece camera (Aunet Scientific Products, Perth, WA). Cells were stained with trypan blue to assess viability and counter-strained with India ink to assess cell and capsule size. Cell size was determined with ImageJ 1.44p by measuring the diameter of the cell, and capsule size was measured by subtracting the cell size from the diameter of the cell and capsule. Cell volume was calculated based on the average cell radius, using the formula 4/3πr^3^. PASW Statistics 17 (SPSS Inc., version 17) was used to detect differences in cell and capsule sizes by the Independent-Sample *t*-test.

### Protein extraction

*C. gattii* strain V5 was grown in 25 mL of YPD broth at 37°C, 200 rpm to mid-log phase (∼8×10^5^ viable cells/mL) and treated with FLC at a concentration of 20 µg/mL. Cells were harvested at 3, 4 and 6 h post treatment by centrifugation at 4,000 *g* for 10 minutes at 4°C, and washed twice in 20 mL of ice-cold distilled water supplemented with 0.5× protease inhibitors (Roche Applied Science, Sydney, NSW, Australia). The cell pellets were resuspended in extraction buffer (7 M urea, 2 M thiourea, 1% C7bzO, 40 mM Tris and 50 mM lithium chloride) supplemented with 1× protease inhibitors, to a concentration of 0.3 mL wet volume of cells/mL buffer, and were disrupted using 2.0 mm glass beads and a Mikro Dismembrator II (B. Braun Biotech, Allentown, PA) at full speed for 5 minutes. Cell lysates were treated with a final concentration of 10 mM tributylphosphine (Sigma-Aldrich, Castle Hill, New South Wales) and acrylamide (Bio-Rad Laboratories, Inc, Hercules, CA, USA) to reduce and alkylate disulfide bonds simultaneously, followed by sonication with a Misonix Sonicator 3000 ultrasonic probe (Misonix, NY, USA) for 1 minute at full power, and cell breakage was checked under the microscope to ensure complete cell lysis. Samples were cooled on ice for 30 seconds and incubated at room temperature for 60 minutes. Following incubation, the cell-free supernatants were collected by centrifugation at 16,000 *g* for 10 minutes and transferred to acetone (Sigma, St Louise, MO, USA) for protein precipitation at room temperature for 60 minutes. Precipitated proteins were collected by centrifugation at 3,000 *g* for 10 minutes and suspended in SDS sample buffer (2% SDS, 40 mM Tris-HCl, pH 6.8, 10% glycerol and 0.1% bromophenol blue). Protein concentration was determined using bovine serum albumin standards in a gel-based assay, and the samples were heated at 90°C for 5 minutes prior to one-dimensional gel electrophoresis. PASW Statistics 17 was used to detect differences in protein yields among samples by the Paired-Sample *t*-test. Three biological and three technical replicates were conducted for each sample to ensure reproducibility. Since the technical replicates from the 3 h samples had a high level of reproducibility, and multiple time points were selected for the study, only the three biological replicates were analyzed for the 4 and 6 h samples.

### One-dimensional (1D) gel electrophoresis

Approximately 120 µg of extracted protein was loaded onto a 12-well Criterion XT precast polyacrylamide gel with 4–12% Bis-Tris (Bio-Rad, Hercules, CA, USA), alongside a Precision Plus Protein unstained standard molecular weight marker (Bio-Rad, Hercules, CA, USA). The protein samples were separated by electrophoresis in MES running buffer at 160 V until the dye front ran off the gels. Gels were then fixed in a solution containing 40% methanol and 10% acetic acid for 30 minutes, stained overnight with Flamingo quantitative fluorescent gel stain (Bio-Rad Laboratories, Inc, Hercules, CA, USA) and imaged at medium intensity with 50 µm pixel size resolution using a Bio-Rad Pharos FX Plus molecular imager (Bio-Rad Laboratories, Inc, Hercules, CA, USA). Lanes containing separated proteins were excised from the stained gels and sliced evenly into twenty fractions. Each fraction was finely macerated, and gel pieces from each fraction were subjected to trypsin in-gel digestion for mass spectrometry as described previously [34].

### Protein identification and validation

Mass spectra for each sample fraction were recorded on a QSTAR Elite hybrid quadrupole-time-of-flight mass spectrometer (Applied Biosystems/MDS Sciex, Carlsbad, CA, USA), and MS/MS data generated for the twenty fractions were merged using Mascot Daemon (Matrix Science, London, England, Thermo). Data were then searched against the LudwigNR databases (ftp://ftp.ch.embnet.org/pub/databases/nr_prot) to identify proteins using the Mascot algorithm (version 2.1.0) provided by the Australian Proteomics Computational Facility (http://www.apcf.edu.au/). The search parameters were as follows: propionamide modifications with possible oxidation of methionine, trypsin digest with three missing cleavages, peptide mass tolerance of ±100 ppm, and MS/MS mass tolerance of ±0.2 Da. A significant probability-based Mowse score (*p*<0.05) was used as the criterion for correct protein identification. Searches against a decoy database were performed to validate searches of MS/MS data.

MS/MS data and protein identifications assessed by Mascot were further validated and analysed using Scaffold (version 2.01.02, Proteome Software Inc., Portland). Protein identifications that had >95% protein probability with at least two unique peptides, as specified by the Protein Prophet algorithm [72], and >95% peptide probability, based on the Peptide Prophet algorithm [73], were accepted. Proteins that contained similar peptides and could not be differentiated based on MS/MS analysis alone were grouped to satisfy the principles of parsimony.

Proteins that were identified in all three biological samples were included for the analysis. Among all the identified proteins, several did not appear to belong to *Cryptococcus* species. However, sequence alignment of these non-cryptococcal proteins with the closely related cryptococcal proteins revealed a high sequence similarity (data not shown). As these proteins met all the filter criteria and were found in all three biological samples, they were included in the analysis. Biological functions of the identified proteins were obtained from the ExPASy Proteomics Server (http://ca.expasy.org/) based on *Cryptococcus* species or other fungal pathogens. Spectral count results of the identified proteins were normalised to reduce technical bias and produce high quality counts for quantitative analysis [74]. Differentially expressed proteins determined from normalised spectrum counts showing an at least 2-fold change in abundance in at least two of the three biological samples were analysed to detect differences in abundance by the *t*-Test provided in Scaffold. The Levene Statistic was used to assess differences in homogeneity between samples.

### Protein interaction networks using GEOMI

Proteins that were differentially regulated in response to FLC were further examined to study protein-protein interactions. GEOmetry for Maximum Insight (GEOMI; http://www.systemsbiology.org.au/downloads_geomi.html) [75] was used to visualize cellular interaction networks using two high-quality yeast protein-protein interaction metadatasets collated by Bertin *et al.* [12] and Yu *et al.* [11]. To generate the networks, yeast homologs of cryptococcal proteins that were differentially expressed over the three time points (Table S2) were first identified using the Clusters of Orthologous Groups (http://www.ncbi.nlm.nih.gov/COG/) [76] or the *Saccharomyces* Genome Database (http://www.yeastgenome.org/). A match with an E-value of less than 10^−9^ was considered acceptable (Table S3). Due to the differences between *Cryptococcus* and *S. cerevisiae* as well as current experimental limitations of detecting protein-protein interactions, not all cryptococcal proteins had a yeast homolog and not all the yeast orthologs were covered in the yeast network.

To minimise visual complexity, networks were generated by seeding with yeast homologs, and extending to show the most direct connections (shortest path) with known protein-protein interactions. Network layout algorithms such as Spring Force, Repulsion Force, Origin Force and Planar Force parameters were then optimised to filter out undesired details and enhance the visualisation in 3D. Proteomic data were mapped onto the drug response networks to contextualise and highlight different aspects of the yeast homologs. This allowed the networks to be analysed in two ways. One was based on the protein expression of differentially regulated proteins in response to FLC, which allows visualisation of the change in protein expression over time; the other was based on Gene Ontology (GO) terms that identifies the functions of the proteins in the interactome network, and can be used to deduce the cellular context of the differentially expressed proteins. The differentially expressed proteins were highlighted (by larger size) to distinguish them from their interacting partners in both networks, and a different color was assigned to each biological process in the latter network, as annotated by the Gene Ontology term.

## Supporting Information

Figure S1 **Larger version of drug response networks shown in** **Figure 4** **.**
(PDF)

Figure S2 **Larger version of drug response networks shown in** **Figure 5** **.** Black arrows indicate essential proteins; red arrows show proteins involved in coordinated process in drug response (see Figure 4 for expression of these proteins).
(PDF)

Table S1 ***Cryptococcus gattii*** **proteins differentially expressed during growth with and without FLC.**
(PDF)

Table S2 **Differential expression of** ***Cryptococcus gattii*** **proteins over time in response to FLC.**
(PDF)

Table S3 **Identification of yeast homologs for cryptococcal proteins differentially expressed in response to FLC.**
(PDF)
