# Supplementary figures and images for: Time-Course Proteome Analysis Reveals the Dynamic Response of Cryptococcus gattii Cells to Fluconazole
Source: PLoS One. 2012 Aug 6;7(8):e42835. doi: 10.1371/journal.pone.0042835 (PMC3412811; doi:10.1371/journal.pone.0042835)

**A: 3 h**

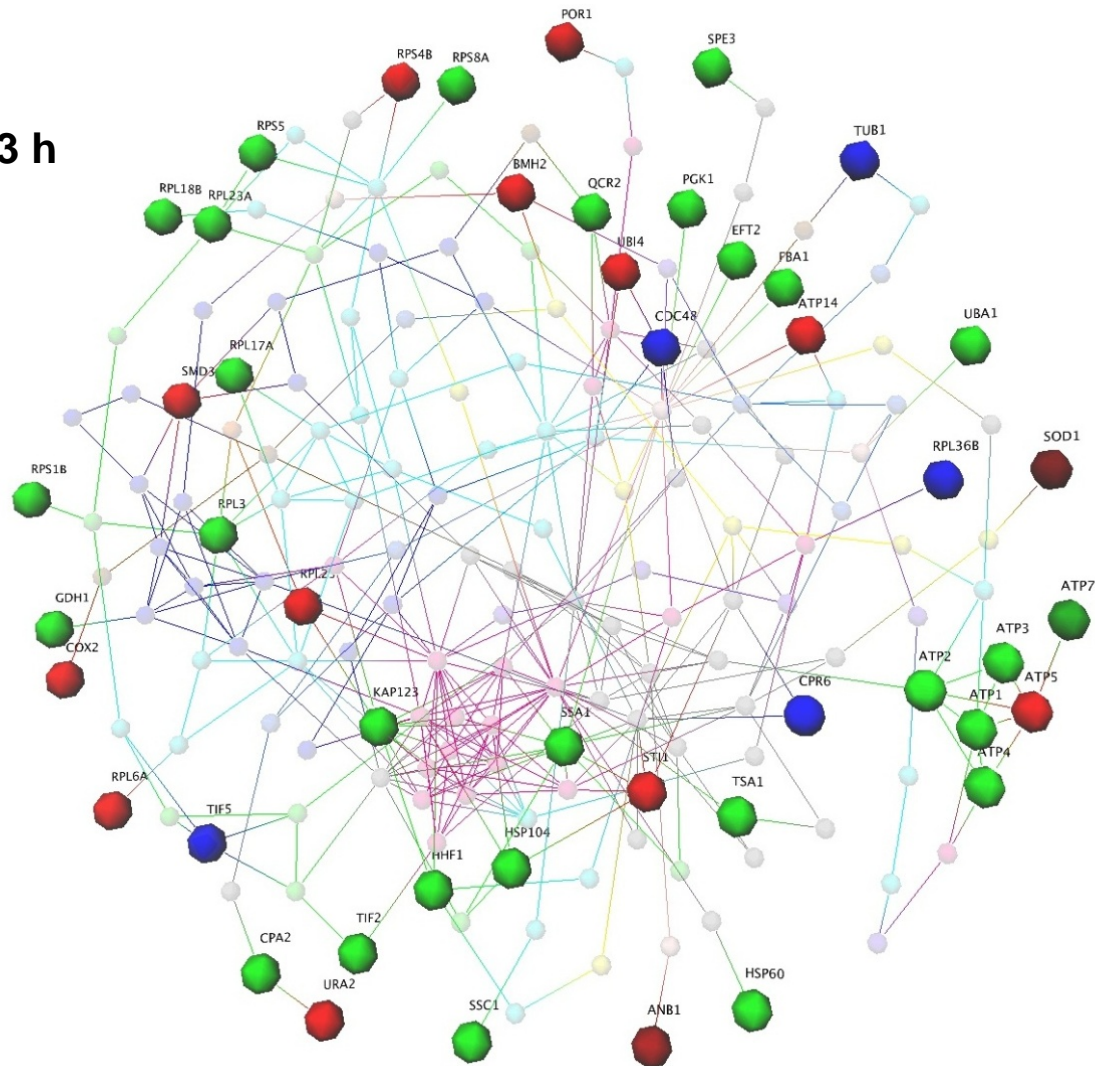

**B: 4 h**

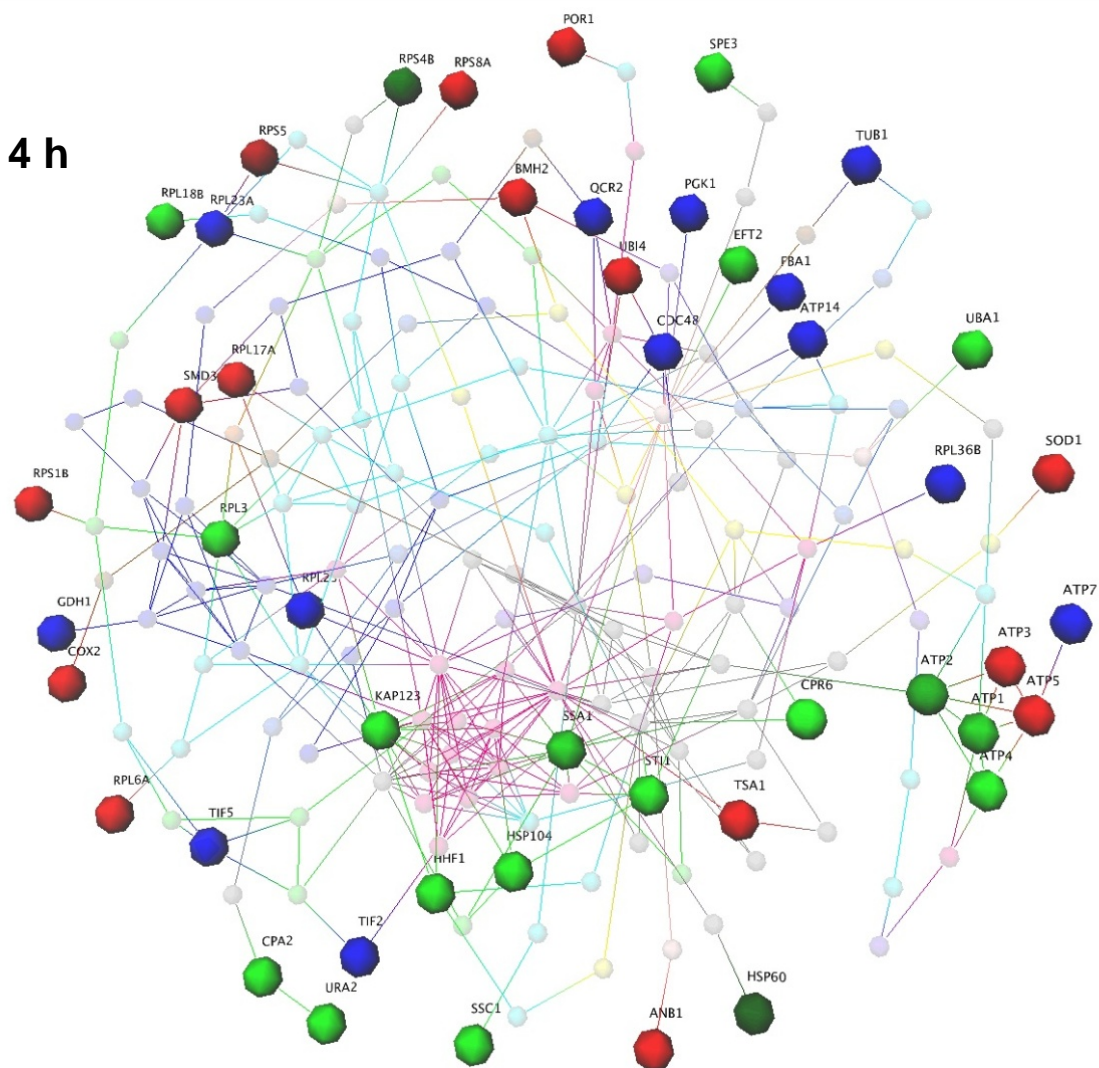

Supplement: Figure S1 — Larger version of drug response networks shown in Figure 4 . (PDF) [file pone.0042835.s001.pdf]
