# Supplementary material for: Time-Course Proteome Analysis Reveals the Dynamic Response of Cryptococcus gattii Cells to Fluconazole
Source: PLoS One. 2012 Aug 6;7(8):e42835. doi: 10.1371/journal.pone.0042835 (PMC3412811; doi:10.1371/journal.pone.0042835)
Supplement: Figure S2 — Larger version of drug response networks shown in Figure 5 . Black arrows indicate essential proteins; red arrows show proteins involved in coordinated process in drug response (see Figure 4 for expression of these proteins). (PDF) [file pone.0042835.s002.pdf]

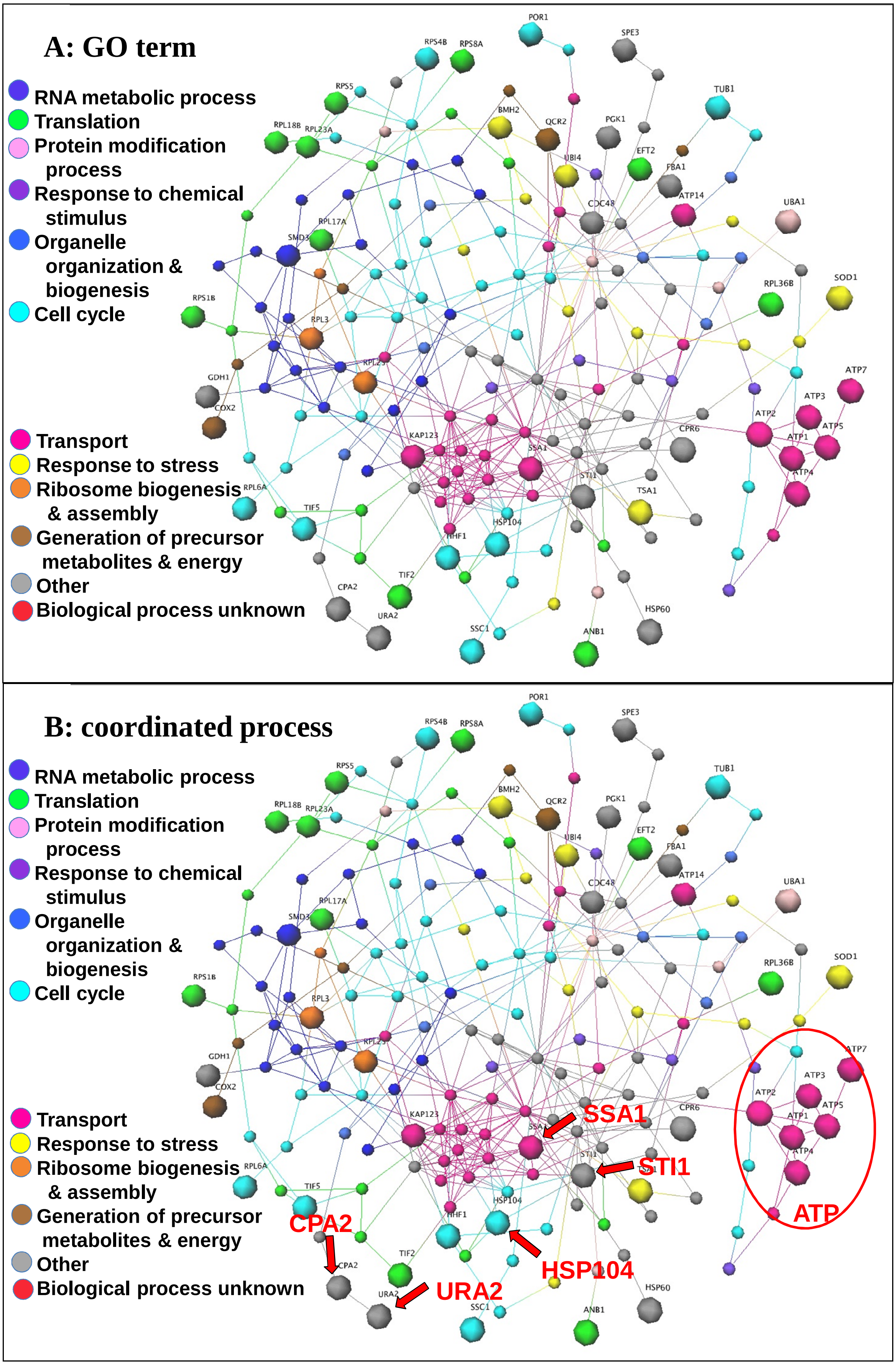
A: GO term
RNA metabolic process
Translation
Protein modification process
Response to chemical stimulus
Organelle organization & biogenesis
Cell cycle
Transport
Response to stress
Ribosome biogenesis & assembly
Generation of precursor metabolites & energy
Other
Biological process unknown
POR1
SPE3
RPS4B
RPS8A
RPS5
TUB1
BMH2
QCR2
PGK1
RPL18B
RPL23A
EFT2
FBA1
UBI4
ATP14
CDC48
UBA1
RPL17A
SMD3
RPL36B
SOD1
RPS1B
RPL3
GDH1
COX2
ATP7
ATP3
ATP2
ATP5
ATP1
ATP4
KAP123
SSA1
CPR6
STI1
TSA1
RPL6A
TIF5
HSP104
HHF1
TIF2
CPA2
URA2
SSC1
ANB1
HSP60
B: coordinated process
SSA1
STI1
CPA2
HSP104
URA2
ATP

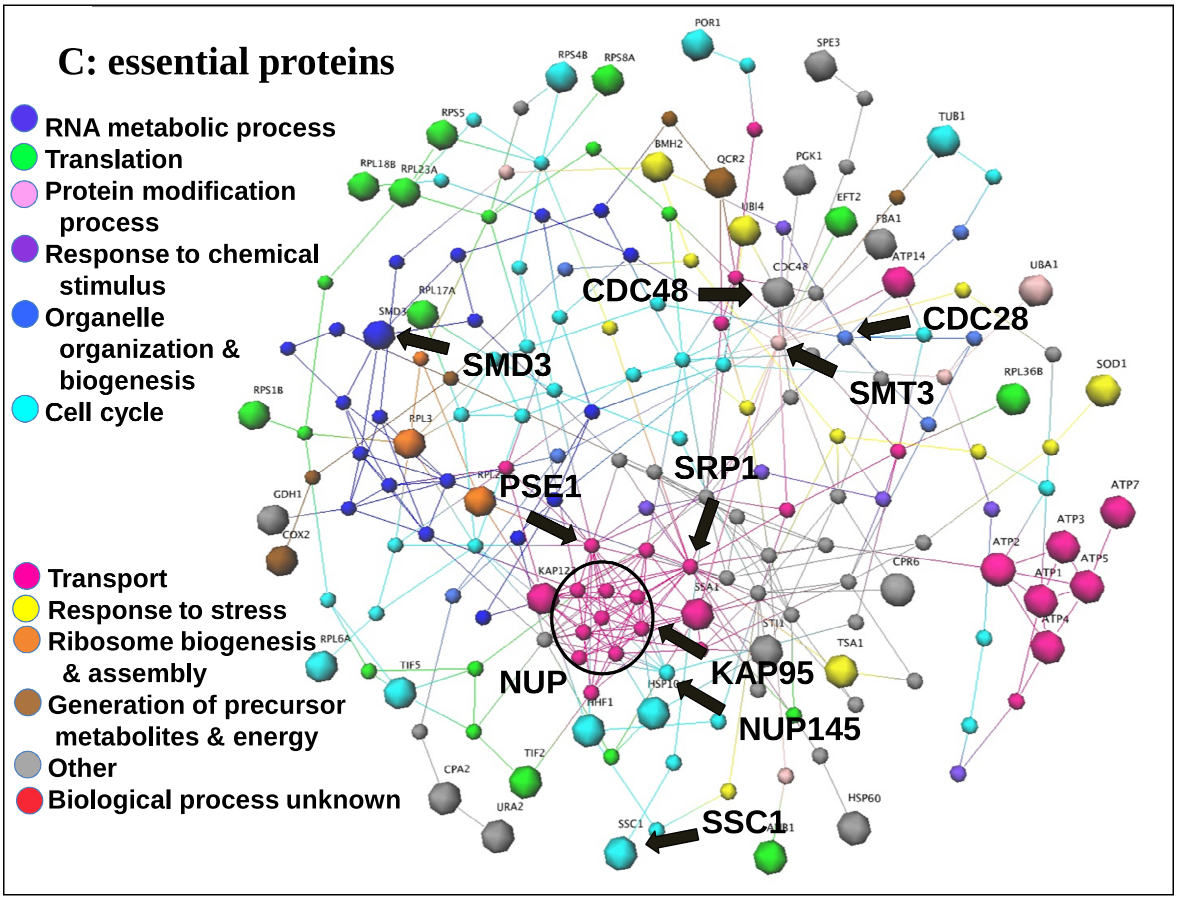

Figure S2: Larger version of drug response networks shown in Figure 5. Black arrows indicate essential proteins; red arrows show proteins involved in coordinated process in drug response (see Figure 4 for expression of these proteins).
