# Supplementary material for: Time-Course Proteome Analysis Reveals the Dynamic Response of Cryptococcus gattii Cells to Fluconazole
Source: PLoS One. 2012 Aug 6;7(8):e42835. doi: 10.1371/journal.pone.0042835 (PMC3412811; doi:10.1371/journal.pone.0042835)
Supplement: Table S1 — Cryptococcus gattii proteins differentially expressed during growth with and without FLC. (PDF) [file pone.0042835.s003.pdf]

Table S1: *Cryptococcus gattii* proteins differentially expressed during growth with and without FLC

Protein name (putative)	Accession #	Function description[1]	Fold change (compared to 3 h)[2]			
			Untreated		FLC-treated	
			4 h	6 h	4 h	6 h
**Immune/stress response**						
Cation-transporting ATPase	Q55SS2	Response to stress; protein folding	--	I	--	--
Hsp70	Q55UJ0	Heat shock protein 70 family; involved in pleiotropic drug resistance via sequential activation of PDR1 and PDR5	--	I	--	--
Heat shock protein 70	Q4P1U5	Heat shock protein 70 family; ATP binding	N	N	--	--
Chaperone	Q5KQ06	Chaperone; assist in protein folding/unfolding and assembly/disassembly	N	+2*	S	+1.5
UDP-glucose dehydrogenase	Q55MH3	Nucleotide-sugar synthesis; essential for growth at 37°C and for capsule biosynthesis	+2.4	+2.2	--	I
**Signal transduction**						
14-3-3 protein	Q5K8Z6	Regulatory protein; signal transduction; play important roles in a wide range of vital regulatory processes	S	+3*	N	+1.8
Rab11 protein	Q55SK4	Small GTPase mediated signal transduction; involved in the exocytic pathway; mediate intra-Golgi traffic or the budding of post-Golgi vesicles from the trans-Golgi	--	I	--	--
RAN small monomeric GTPase	Q5KGN7	Small GTPase mediated signal transduction	--	I	--	--
**Ribosomal proteins**						
40S ribosomal protein S13	Q5KIJ0	Component of ribosome	+1.5	-2*	S	+2
60S ribosomal protein L20	Q55KZ4	Component of ribosome; translation	--	I	--	--
60s ribosomal protein l30-1 (L32)	Q5KPM8	Component of ribosome; translation	S	N	--	--
60s ribosomal protein l38 (Yml38)	Q5KJU6	Component of ribosome; translation	S	N	--	--
Ribosomal protein s5-1	Q5K947	Component of ribosome; translation; essential for viability	+3	-2.5*	+2.3	+1.6
Ribosomal protein S11	Q5KNH2	Component of ribosome; translation	--	I	--	--
Ribosomal protein S17	Q5KIH4	Component of ribosome; translation	--	I	--	--
Ribosomal protein L13	Q5K7W8	Component of ribosome; translation	S	+1.5	--	--
Ribosomal protein L15	Q5KJD4	Component of ribosome; translation	--	I	--	--

Ribosomal protein l34-b	Q5KN25	Component of ribosome	-2	-2*	I	--
Ribosomal protein L27	Q55YU7	Component of ribosome	S	+3*	--	--
Ribosomal protein L36	Q5KEE1	Component of ribosome; translation	--	I	--	I
Ribosomal protein of the small subunit	Q5KGW0	Component of ribosome; translation; rRNA binding	I	I	--	--
Small subunit ribosomal protein 14	Q5KFT0	Component of ribosome; translation	S	+2	--	--
40S ribosomal protein S4	Q5KNK2	Component of ribosome; translation; RNA binding	+2	+2	-1.8	-2.5*
Large subunit ribosomal protein L3	Q5K9E3	Component of ribosome; translation	N	+1.5	--	I
Ribosomal protein l5-b	Q5KJU2	Component of ribosome; translation; 5S rRNA binding	S	+2	I	--
Ribosomal protein l23	Q5K959	Component of ribosome; translation	S	+2	--	I
Ribosomal protein of the large subunit	Q5KL89	Component of ribosome; translation	S	+3	S	-3.5*
**Sugar/lipid metabolism**						
6-phosphofructokinase	Q5KAN4	Glycolysis; convert D-fructose-6-phosphate into D-fructose 1,6-biphosphate	--	I	--	I
Acetyl-CoA carboxylase	Q55QT6	Fatty acid biosynthetic process	-2.2	N	--	--
Isocitrate dehydrogenase (NAD+)	Q5KAD7	TCA cycle; Isocitrate dehydrogenase (NAD+) activity	--	I	--	--
Fatty acid synthase alpha subunit	Q2HYU3	Fatty acid biosynthesis; magnesium ion; bindingtransferase and synthase activities	N	+3	--	--
Fructose-bisphosphate aldolase	Q5KMW2	Glycolysis; fructose-bisphosphate aldolase activity	S	+3*	--	I
Glucose-6-phosphate isomerase	Q55Y45	Glycolysis; glucose-6-phosphate isomerase activity	--	I	--	--
Glutamine-fructose-6-phosphate transaminase (Isomerizing)	Q5KII0	Carbohydrate biosynthetic process	--	--	--	I
Glyceraldehyde-3-phosphate dehydrogenase (Fragment)	B3TGN5	Glycolysis; glyceraldehyde-3-phosphate dehydrogenase (phosphorylating) activity	N	-1.8	--	--
NADPH dehydrogenase 2	Q55N61	Oxidoreductase activity; may be involved in sterol metabolism	--	--	--	I
Oxoglutarate dehydrogenase (Succinyl-transferring)	Q5KMH0	Glycolysis; oxoglutarate dehydrogenase (succinyl-transferring) activity; energy production in TCA cycle	--	I	--	I
Transketolase	Q55T38	Catalytic activity in the pentose phosphate pathway; needed for synthesis of aromatic amino acids	--	I	--	--
Fatty acid synthase complex protein	Q5KG98	Fatty acid biosynthesis	N	+2	--	I
Phosphoglucomutase	Q5K7B5	Carbohydrate metabolic process; hexose metabolism	--	--	--	I
Pyruvate kinase	Q5KKG5	Glycolysis; carbohydrate degradation	N	+2	--	I
**Nuclear proteins**						

Curved DNA-binding protein homolog	Q5KJ40	DNA binding; hydrolase activity	S	N	--	I
Histone H4	Q5K8H5	Core component of nucleosome; play a central role in transcription regulation, DNA repair, DNA replication and chromosomal stability	N	+2	S	-2*
Small nuclear ribonucleoprotein E	Q5KBD5	Nucleic acid binding	--	--	S	S
**Protein/amino acid metabolism**						
Aminoadipate-semialdehyde dehydrogenase	Q5KEK6	Lysine biosynthesis; cofactor/phosphopantetheine bindings	--	I	--	--
Aspartate carbamoyltransferase	Q5KNM2	Pyrimidine base biosynthesis, glutamine metabolism	S	+2.5*	S	+3.6*
ER-associated protein catabolism-related protein	Q5KHB5	Protein folding; unfolded protein binding; ATP binding	--	I	--	--
Eukaryotic translation initiation factor 3 subunit A	Q5KGK5	Protein biosynthesis; translation; together with other initiation factors to stimulate binding of mRNA and methionyl-tRNAi to the 40S ribosome	S	N	--	--
Eukaryotic translation initiation factor 3 subunit C	Q5KH72	Protein biosynthesis; translation; together with other initiation factors to stimulate binding of mRNA and methionyl-tRNAi to the 40S ribosome	S	-2*	--	--
Eukaryotic translation initiation factor 5C homolog	Q5KI79	Translation initiation factor activity	--	I	--	I
FK506-binding protein 1 (Peptidyl-prolyl cis-trans isomerase)	O94746	Protein folding; catalyse the cis-trans isomerization of proline imidic peptide bonds in oligopeptides	--	I	S	S
FK506-binding protein 4 (Peptidyl-prolyl cis-trans isomerase)	Q5KIJ5	Protein folding; catalyze the cis-trans isomerization of proline imidic peptide bonds in oligopeptides	S	+1.5	--	--
GrpE protein	Q55K54	Protein folding and transport into mitochondrial matrix	S	N	--	--
Homocitrate synthase	Q5KIZ5	Lysine biosynthesis; transfer acyl groups	--	I	--	I
Homoisocitrate dehydrogenase	Q55U60	Lysine biosynthesis; act on the CH-OH group of donors, NAD or NADP as acceptor	--	I	--	--
Isoleucine-tRNA ligase	Q5KPM2	Isoleucyl-tRNA aminoacylation; ATP binding	S	S	--	I
Mitochondrial processing peptidase beta subunit, mitochondrial (Beta-mpp)	Q5KED7	Proteolysis; metalloendopeptidase activity; zinc ion binding	S	+3	--	--
MMS2	Q5KA71	ATP-dependent peptidase activity; serine-type endopeptidase activity	--	I	--	I
Nascent polypeptide-associated complex subunit alpha	Q5K8B4	Protein transport; regulation of transcription	S	N	--	--
Nascent polypeptide-associated	Q5KCH5	Protein transport; regulation of transcription	S	+2.5	--	--

complex subunit beta						
Proline-tRNA ligase	Q55YK1	Prolyl-tRNA aminoacylation	S	+2	--	--
Protein TIF31 homolog	Q5K7G8	Translation initiation factor	S	+1.5	--	--
Serine hydroxymethyltransferase	Q5KAU8	One-carbon metabolism; serine metabolic process	S	-3	--	--
T-complex	Q55K28	Protein folding; unfolded protein binding; ATP binding; required for the assembly of actin and tubulins	S	S	--	--
T-complex protein 1, theta subunit (Tcp-1-theta)	Q5KKB4	Protein folding in cytoplasm; chaperone; unfolded protein binding; ATP binding	--	I	--	--
Translation initiation factor	Q5KMN3	Protein synthesis; GTP binding	--	I	--	--
Adenosylhomocysteinase	Q5KJ87	Homocysteine biosynthesis; 1 carbon metabolism	S	N	--	I
Arginine-6 protein	Q55QU3	Arginine biosynthesis, mitochondrion	--	--	--	I
Polyubiquitin	O35079	Protein modification process	+1.4	+1.6	+1.5	-4*
**Plasma membrane proteins**						
ATP-binding cassette transporter	A3QWE5	Protein transport and binding; integral into membrane; coupled to transmembrane movement of substances	--	I	I	--
Cassette (ABC) transporter	Q5KJ82	Protein transport and binding	--	I	--	--
Isoprenoid biosynthesis-related protein	Q5KG83	Isoprene biosynthesis to form lanosterol	--	I	--	I
**Cytoskeleton proteins**						
Actin lateral binding protein	Q5KF76	Bind to and stabilize actin cables and filaments	--	I	--	--
Alpha tubulin	Q5KM62	Microtubule-based movement; protein polymerization; structural molecular activity	--	I	--	I
Beta1-tubulin	Q5KKE7	Microtubule-based movement; protein polymerization	S	N	--	--
Actin	Q5KP06	Cytoskeleton; involved in various types of cell motility	S	+1.5	--	I
**Miscellaneous**						
Aldehyde reductase	Q55K68	Oxidoreductase activity; alcohol dehydrogenase (NADP+) activity	--	I	--	I
C1-tetrahydrofolate synthase	Q55NZ3	Folic acid and derivative biosynthesis; involved in single carbon metabolism; required for biosynthesis of purines, thymidylate, methionine, and histidine	S	N	S	N
Chaperone regulator	Q5KLR7	Regulation of the HSP90 and HSP70 functions; protein translocation across membranes	S	+2	--	--
Clathrin heavy chain 1	Q5KA29	Intracellular protein transport; vesicle-mediated transport; clathrin coat	--	I	--	--

Cytochrome c oxidase subunit 2	Q85SZ4	Respiratory electron transport chain; form the functional core of the enzyme complex; electron transfer	--	I	N	+1.5
Cytochrome c oxidase subunit V	Q5K946	Electron transfer in respiratory chain	S	N	--	--
Dihydrolipoyl dehydrogenase	Q5Y229	Cell redox homeostasis; dihydrolipoyl dehydrogenase activity	I	I	--	--
Hydrogen-transporting ATP synthase	Q5KJV2	ATP hydrolysis coupled proton transport	--	I	S	S
Inorganic diphosphatase	Q5KHF9	Phosphate metabolic process	S	+1.6	--	--
Mitochondrial C1-tetrahydrofolate synthase	Q55NZ3	Folic acid and derivative biosynthesis; ATP binding	S	N	--	--
Mitochondrial import inner membrane translocase subunit TIM8	Q5KFM0	Intracellular protein transmembrane transport; protects the hydrophobic precursors from aggregation and guide them through the mitochondrial intermembrane space	S	+2	--	--
Mitochondrial import inner membrane translocase subunit TIM10	Q55U43	Intracellular protein transmembrane transport; protects the hydrophobic precursors from aggregation and guide them through the mitochondrial intermembrane space	--	I	S	S
NADH dehydrogenase 10.5K chain	Q5KCH4	Respiratory chain complex 1	--	I	--	--
NADH-ubiquinone oxidoreductase 30.4 kDa subunit	Q5KF23	Electron transfer in respiratory chain	S	N	--	--
Nucleoside-diphosphate kinase	Q55KK1	Nucleoside diphosphate kinase activity	S	N	--	--
Pre-mRNA splicing factor	Q55XU9	mRNA processing	--	I	--	--
RNA-binding G protein effector homolog	Q55N75	Mating response pathway; mainly associated with nuclear envelope and ER; interact in mRNA-dependent manner with translating ribosomes	--	I	--	--
Transferase	Q5KHG0	Transferase activity; required for xanthine utilization and for optimal utilization of guanine	--	I	--	--
U6 snRNA-associated Sm-like protein LSm2 homolog	Q55VD7	mRNA processing	--	I	--	--
Ubiquinol-cytochrome C reductase iron-sulfur subunit, mitochondrial	Q5KGU5	Electron transport; respiratory chain in mitochondrion	--	I	--	--
ATP synthase complex subunit H	Q5KIZ7	ATP synthesis; hydrogen ion transport	S	N	S	S
Complex 1 protein	Q5KNR5	Transfer of electrons from NADH to the respiratory chain	S	N	S	S
Electron transporter, transferring electrons within CoQH2-cytochrome c reductase complex	Q5KFT4	Respiratory chain; electron carrier activity; heme/iron ion bindings	S	N	I	--
NADH dehydrogenase	Q5KN57	Oxidoreductase activity; FAD binding	--	I	--	I

NADH-ubiquinone oxidoreductase	Q55XC1	ATP synthesis coupled electron transport	--	--	--	I
Polyadenylate-binding protein, cytoplasmic and nuclear	Q5KBW2	mRNA processing/transport; regulation of translation; an important mediator of the multiple roles of the poly(A) tail in mRNA biogenesis, stability and translation	N	N	--	I
Rab/GTPase	Q5KK41	Protein transport; essential for exocytosis; may regulate polarized delivery of transport vesicles to the exocyst at the plasma membrane	--	I	S	S
Succinate dehydrogenase flavoprotein subunit	Q5KBA0	Electron transport chain; FAD binding	--	--	--	I
Ubiquinol-cytochrome C reductase complex core protein 2	Q5K8U4	Electron transport; metalloendopeptidase activity; zinc ion binding	S	N	--	I
UTP-glucose-1-phosphate uridylyltransferase	Q5KKA5	Nucleotidyltransferase activity; catalyse the reversible formation of UDP-Glc from glucose 1-phosphate and UTP	S	N	--	I
**Unknown**						
Cytoplasm protein	Q5KAL4	Unknown	S	N	--	--
Cytoplasm protein	Q55ZC0	Unknown	--	--	--	I
Predicted protein, ATP-dependent	A0CAI2	Unknown	--	--	I	--
Uncharacterized protein	Q5KJK9	Unknown	--	--	S	S
Uncharacterized proteins	Q55SN6	Unknown	--	--	S	S
Uncharacterized protein	Q55PA2	Unknown	--	--	S	S

[1] Searches based on genome data for *Cryptococcus* species, or for other fungi if not available.

[2] Based on ratio of normalised spectrum counts. I: induced (only present at that time point and not at other time points); S: suppressed (not present at that time point but found at other time points); N: no change; -- : protein absent at that time point. * indicates significant difference ($p < 0.05$).
