# Supplementary material for: Time-Course Proteome Analysis Reveals the Dynamic Response of Cryptococcus gattii Cells to Fluconazole
Source: PLoS One. 2012 Aug 6;7(8):e42835. doi: 10.1371/journal.pone.0042835 (PMC3412811; doi:10.1371/journal.pone.0042835)
Supplement: Table S2 — Differential expression of Cryptococcus gattii proteins over time in response to FLC. (PDF) [file pone.0042835.s004.pdf]

Table S2: Differential expression of *Cryptococcus gattii* proteins over time in response to FLC

Cryptococcus[1]		Trend[2]		
**Protein name (putative)**	**Accession**	**3h**	**4h**	**6h**
**Immune/stress response**				
Chaperone	Q5KQ06	U	S	N
Copper/zinc superoxide dismutase	Q6VTE9	U	U	S
Heat shock protein (HSP60 family)	Q5KLW7	D	D	D
Heat shock protein (HSP70 family)	Q5K8W5	D	D	D
Heat shock protein (chaperone clpA/clpB family)	Q5KPH0	D	S	U
Heat shock protein 70	Q5KKP4	D	D	U
HSP12	Q6TGJ5	U	--	--
Thiol-specific antioxidant protein 1 (Fragment)	Q2QEI6	N	U	U
**Signal transduction**				
14-3-3 protein	Q5K8Z6	U	I	U
G protein beta subunit Gib2	A0AUJ0	D	D	D
**Ribosomal proteins**				
40S ribosomal protein S1	Q5KLL1	D	I	D
40S ribosomal protein S4	Q5KNK2	U	D	D
Ribosomal protein s5-1	Q5K947	D	U	U
40S ribosomal protein S7	Q55NI0	U	U	U
40S ribosomal protein S8	Q5KDJ7	S	I	D
40S ribosomal protein S13	Q5KIJ0	D	S	N
40S ribosomal protein S16	Q5KM68	U	--	D
60s ribosomal protein L1-a	Q5KGE3	S	U	D
60s ribosomal protein l7	Q5KNI6	S	I	D
60s ribosomal protein l17	Q5K6Z0	N	U	D
Ribosomal protein l23	Q5K959	S	--	D
Ribosomal protein L36	Q5KEE1	--	--	D
Large subunit ribosomal protein L3	Q5K9E3	S	S	D
Ribosomal protein of the large subunit	Q5KL89	U	--	D
Ribosomal protein of the large subunit	Q5KNE0	U	U	S
Ribosomal protein L6	Q5KGM8	U	I	U
Ribosomal protein l9	Q5KQ70	U	U	D
Ribosomal protein L18	Q5KIC5	N	D	D
Ribosomal protein L24 (L30)	Q5KDS7	U	D	D

Ribosomal protein l34-b	Q5KN25	D	U	S
Ribosomal protein L35	Q5KN73	U	D	S
Ribosomal protein S18	Q5KA46	D	N	S
Ribosomal protein S19	Q5KJL6	N	N	D
Ribosomal protein s21	Q5KI51	U	U	N
**Sugar/lipid metabolism**				
6-phosphogluconate dehydrogenase, decarboxylating	Q5K9R3	S	--	D
ATP-citrate synthase	Q5KAR2	D	U	U
Citrate synthase	Q5KQ45	U	U	D
Enolase	Q5KLA7	S	D	D
Fatty-acid synthase complex protein	Q5KG98	S	S	D
Fructose-bisphosphate aldolase	Q5KMW2	S	--	D
Glutamate dehydrogenase (NADP+)	Q5KL32	S	--	D
Glyceraldehyde-3-phosphate dehydrogenase	Q9Y8E9	D	D	U
Malate dehydrogenase	Q5KDL9	D	U	N
Phosphoglycerate kinase	Q5KE00	S	--	U
Pyruvate carboxylase	Q55QD4	S	D	D
Transaldolase	Q5K952	D	U	U
UDP-xylose synthase	Q7LJU0	S	S	D
**Nuclear proteins**				
Histone H4	Q5K8H5	N	S	D
**Protein/amino acid metabolism**				
5-methyltetrahydropteroyltriglutamate-homocysteine S-methyltransferase	Q5K9D7	N	D	U
Aspartate carbamoyltransferase	Q5KNM2	U	S	D
ATP-dependent RNA helicase Eif4a	Q5KN60	S	--	D
Carbamoyl-phosphate synthase subunit arginine-specific large	Q5K7V3	S	S	D
Elongation factor 1-gamma	Q55ZV5	N	D	U
Eukaryotic translation initiation factor 5C homolog	Q5KI79	--	--	U
Initiation factor 5a (Eif-5a)	Q5KHT0	U	I	D
MMS2	Q5KA71	--	--	U
Peptidyl-prolyl cis-trans isomerase D	Q5KFV5	--	S	U
Polyubiquitin	O35079	U	U	D
Translation elongation factor 2	Q9HFZ8	D	D	U
Ubiquitin activating enzyme	Q560X2	S	S	U
**Plasma membrane proteins**				
Isoprenoid biosynthesis-related protein	Q5KG83	--	--	U

Plasma membrane H(+)-ATPase	Q9UR20	D	U	U
Plasma membrane H(+)-ATPase 1	O74242	D	N	U
**Cytoskeleton proteins**				
Alpha tubulin	Q5KM62	--	--	D
**Miscellaneous**				
ATP synthase	Q5KL26	S	N	D
ATP synthase complex subunit H	Q5KIZ7	U	--	S
ATP synthase delta subunit	Q5KIE2	U	I	S
ATP synthase gamma chain	Q55SW7	S	U	S
ATP synthase subunit beta	Q5KFU0	D	D	U
ATP synthase subunit alpha	Q5KFB9	D	D	U
Chimeric spermidine synthase/saccharopine dehydrogenase	Q6RXX2	S	S	D
Complex 1 protein	Q5KNR5	U	--	S
Cytochrome c oxidase subunit 2	Q85SZ4	I	I	U
Electron carrier	Q5KNC7	U	U	S
Importin beta-4 subunit	Q5KFR0	S	D	D
Inorganic phosphate transporter	Q5K756	N	U	U
NADH dehydrogenase	Q5KN57	--	--	D
Pre-mRNA splicing factor	Q5KLG7	U	I	S
Structural molecule	Q5KLP2	D	--	D
Peripheral-type benzodiazepine receptor homolog	Q5K6Y4	U	U	U
Ubiquinol-cytochrome C reductase complex core protein 2	Q5K8U4	S	--	U
Voltage-dependent ion-selective channel	Q5KJP2	U	U	N

[1] Obtained from Uniprot (http://www.uniprot.org/).

[2] Based on ratio of normalised spectrum counts in FLC treated versus untreated sample at each time point. U: up-regulated; D: down-regulated; I: induced (present in treated samples only); S: suppressed (present in untreated samples only); N: no change; -- : protein absent in both treated and untreated samples. Actual values are given in Table S3.
