# Supplementary material for: Time-Course Proteome Analysis Reveals the Dynamic Response of Cryptococcus gattii Cells to Fluconazole
Source: PLoS One. 2012 Aug 6;7(8):e42835. doi: 10.1371/journal.pone.0042835 (PMC3412811; doi:10.1371/journal.pone.0042835)
Supplement: Table S3 — Identification of yeast homologs for cryptococcal proteins differentially expressed in response to FLC. (PDF) [file pone.0042835.s005.pdf]

Table S3: Identification of yeast homologs for cryptococcal proteins differentially expressed in response to FLC

Cryptococcal protein[1]		Yeast protein[2]		Fold change[4]		
**Protein name (putative)**	**Accession**	**Gene/ protein name**	**E value[3]**	**3h**	**4h**	**6h**
**Immune/stress response**						
Chaperone	Q5KQ06	STI1/ Hsp90 cochaperone	1.80E-117	+2	S	N
Copper/zinc superoxide dismutase	Q6VTE9	SOD1/ Cytosolic copper-zinc superoxide dismutase	9.50E-51	+1.3	+2.5	S
Heat shock protein (HSP60 family)	Q5KLW7	HSP60/ Tetradecameric mitochondrial chaperonin	5.00E-196	--	+2	--
Heat shock protein (HSP70 family)	Q5K8W5	SSC1/ Hsp70 family ATPase	2.20E-234	-2.8	-1.2	-1.4
Heat shock protein (chaperone clpA/clpB family)	Q5KPH0	HSP104/ Heat shock protein	1.10E-193	-3	-2*	-1.5
Heat shock protein 70	Q5KKP4	SSA1/ ATPase HSP70 family	1.50E-240	-2.5	S	+1.5
HSP12	Q6TGJ5	HSP12/ Plasma membrane localized protein	1.70E-12	-2*	-1.7	+1.2
Thiol-specific antioxidant protein 1 (Fragment)	Q2QEI6	TSA1/ Thioredoxin peroxidase	1.40E-24	+2	--	--
**Signal transduction**						
14-3-3 protein	Q5K8Z6	BMH2/ 14-3-3 protein	3.50E-101	+2	I	+1.5
G protein beta subunit Gib2	A0AUJ0	ASC1/ G-protein beta subunit and guanine nucleotide dissociation inhibitor for Gpa2p	6.10E-88	-2.5	-1.5	-2.6*
**Ribosomal proteins**						
40S ribosomal protein S1	Q5KLL1	RPS1B/ Ribosomal protein 10 (rp10) of the small (40S) subunit	1.10E-74	-3	I	-2
40S ribosomal protein S4	Q5KNK2	RPS4B/ Component of 40S subunit	5.10E-100	+3	-1.3	-2.2*
40s ribosomal protein s5-1	Q5K947	RPS5/ Component of 40S subunit	2.60E-71	-2	+1.6	+1.5
40S ribosomal protein S7	Q55NI0	RPS7B/ Component of the small (40S) ribosomal subunit	4.20E-48	+1.3	+1.6	+3.5
40S ribosomal protein S8	Q5KDJ7	RPS8A/ Component of the small (40S) ribosomal subunit	3.00E-63	S	I	-2.5
40S ribosomal protein S13	Q5KIJ0	RPS13/ Component of 40S subunit	9.00E-55	-2	S	N
40S ribosomal protein S16	Q5KM68	RPS16B/ Component of the small (40S) ribosomal subunit	1.30E-46	+2	--	-2
60s ribosomal protein L1-a	Q5KGE3	RPL1B/ N-terminally acetylated protein component of the large (60S) ribosomal subunit	2.50E-66	S	+1.5	-2
60s ribosomal protein l7	Q5KNI6	RPL7A/ Component of the large (60S) ribosomal subunit	4.00E-59	S	I	-2
60s ribosomal protein l17	Q5K6Z0	RPL17A/ Component of the large (60S) ribosomal subunit	2.00E-43	N	+2	-2
60s ribosomal protein l23	Q5K959	RPL23A/ Component of 60S subunit	4.60E-58	S	--	-3
60S ribosomal protein L36	Q5KEE1	RPL36B/ Component of 60S subunit	7.20E-14	--	--	-2
Large subunit ribosomal protein L3	Q5K9E3	RPL3/ Component of 60S subunit	2.70E-156	S	S	-2
Ribosomal protein of the large subunit	Q5KL89	RPL25/ Primary rRNA-binding ribosomal protein component of the large (60S) ribosomal subunit	5.00E-38	+2	--	-3

Ribosomal protein of the large subunit	Q5KNE0	RPL31A/ Component of the large (60S) ribosomal subunit	1.10E-31	+3*	+1.4	S
Ribosomal protein L6	Q5KGM8	RPL6A/ N-terminally acetylated protein component of the large (60S) ribosomal subunit	3.90E-36	+3*	I	+2
Ribosomal protein l9	Q5KQ70	RPL9B/ Component of the large (60S) ribosomal subunit	1.20E-50	+2.6	+4	-1.8
Ribosomal protein L18	Q5KIC5	RPL18B/ Component of the large (60S) ribosomal subunit	8.30E-52	N	-2	-1.7
Ribosomal protein L24 (L30)	Q5KDS7	RPL24B/ Ribosomal protein L30 of the large (60S) ribosomal subunit	4.60E-35	+3	-2	-1.5
Ribosomal protein l34-b	Q5KN25	RPL34B/ Component of 60S subunit	1.90E-38	-2*	+2	S
Ribosomal protein L35	Q5KN73	RPL35B/ Component of the large (60S) ribosomal subunit	8.50E-27	+1.7	-2.3	S
Ribosomal protein S18	Q5KA46	RPS18A/ Component of the small (40S) ribosomal subunit	1.60E-55	-2	N	S
Ribosomal protein S19	Q5KJL6	RPS19A/ Component of the small (40S) ribosomal subunit	7.80E-40	N	N	-2
Ribosomal protein s21	Q5KI51	RPS21A/ Component of the small (40S) ribosomal subunit	1.00E-28	+2	+2	N
**Sugar/lipid metabolism**						
6-phosphogluconate dehydrogenase, decarboxylating	Q5K9R3	GND1/ 6-phosphogluconate dehydrogenase (decarboxylating)	1.50E-185	S	-2*	-1.5
ATP-citrate synthase	Q5KAR2	LSC1/ Alpha subunit of succinyl-CoA ligase	2.70E-10	-1.3	+2.8	+5
Citrate synthase	Q5KQ45	CIT1/ Citrate synthase	1.50E-157	+2	+2	-2.5
Enolase	Q5KLA7	ENO1/ Enolase I	9.50E-154	S	-3	-1.8
Fatty-acid synthase complex protein	Q5KG98	FAS1/ Beta subunit of fatty acid synthetase	0	S	S	-3
Fructose-bisphosphate aldolase	Q5KMW2	FBA1/ Fructose 1,6-bisphosphate aldolase	4.20E-128	S	--	-2
Glutamate dehydrogenase (NADP+)	Q5KL32	GDH1/ NADP(+)-dependent glutamate dehydrogenase	5.10E-139	S	--	-3
Glyceraldehyde-3-phosphate dehydrogenase	Q9Y8E9	TDH1/ Glyceraldehyde-3-phosphate dehydrogenase, isozyme 1	3.30E-112	-3	-1.8	+1.6
Malate dehydrogenase	Q5KDL9	MDH1/ Mitochondrial malate dehydrogenase	1.30E-101	-5	+1.5	N
Phosphoglycerate kinase	Q5KE00	PGK1/ 3-phosphoglycerate kinase	2.90E-136	S	--	+2
Pyruvate carboxylase	Q55QD4	PYC2/ Pyruvate carboxylase isoform	0	S	-1.5	-3*
Transaldolase	Q5K952	TAL1/ Transaldolase	1.10E-104	-2	+2	+1.3
UDP-xylose synthase	Q7LJU0	GAL10/ UDP-glucose-4-epimerase	1.00E-06	S	S	-3
**Nuclear proteins**						
Histone H4	Q5K8H5	HHF1/ Histone H4	7.30E-37	N	S	-2
**Protein/amino acide metabolism**						
5-methyltetrahydropteroyltriglutamate-homocysteine S-methyltransferase	Q5K9D7	MET6/ Cobalamin-independent methionine synthase	5.50E-213	N	-2.5	+1.6
Aspartate carbamoyltransferase	Q5KNM2	URA2/ Bifunctional carbamoylphosphate synthetase	0	+2	S	-1.8

		(CPSase)-aspartate transcarbamylase (ATCase)				
ATP-dependent RNA helicase Eif4a	Q5KN60	TIF2/ Translation initiation factor eIF4A	5.80E-131	S	--	-2
Carbamoyl-phosphate synthase subunit arginine-specific large	Q5K7V3	CPA2/ Large subunit of carbamoyl phosphate synthetase	0	S	S	-3
Elongation factor 1-gamma	Q55ZV5	CAM1/ Nuclear protein (has similarity to translational cofactor EF-1 gamma)	6.50E-52	N	-2	+3
Eukaryotic translation initiation factor 5C homolog	Q5KI79	TIF5/ Translation initiation factor eIF5	0.042	--	--	+2
Initiation factor 5a (Eif-5a)	Q5KHT0	ANB1/ Translation elongation factor eIF-5A	7.60E-58	+1.5	I	-2
MMS2	Q5KA71	CDC48/ ATPase	0	--	--	+2.3
Peptidyl-prolyl cis-trans isomerase D	Q5KFV5	CPR6/ Peptidyl-prolyl cis-trans isomerase (cyclophilin)	2.30E-65	--	S	+2*
Polyubiquitin	O35079	UBI4/ Ubiquitin	4.80E-191	+2	+2.8	-3
Translation elongation factor 2	Q9HFZ8	EFT2/ Elongation factor 2 (EF-2)	0	-3	-1.8	+1.6
Ubiquitin activating enzyme	Q560X2	UBA1/ Ubiquitin activating enzyme (E1)	1.50E-288	S	S	+2
**Plasma membrane proteins**						
Isoprenoid biosynthesis-related protein	Q5KG83	ERG20/ Farnesyl pyrophosphate synthetase	1.90E-117	--	--	+3
Plasma membrane H(+)-ATPase	Q9UR20	PMA2/ Plasma membrane H+-ATPase	1.00E-131	-1.4	+1.2	+2.5
Plasma membrane H(+)-ATPase 1	O74242	PMA2/ Plasma membrane H+-ATPase	1.30E-133	-1.8	N	+2.2
**Cytoskeleton proteins**						
Alpha tubulin	Q5KM62	TUB1/ Alpha-tubulin	3.00E-176	--	--	-2
**Miscellaneous**						
ATP synthase	Q5KL26	ATP4/ Subunit b of the stator stalk of mitochondrial F1F0 ATP synthase	3.80E-40	S	N	-2.5
ATP synthase complex subunit H	Q5KIZ7	ATP14/ Subunit h of the F0 sector of mitochondrial F1F0 ATP synthase	0.0054	+2	--	S
ATP synthase delta subunit	Q5KIE2	ATP5/ Subunit 5 of the stator stalk of mitochondrial F1F0 ATP synthase	4.40E-30	+2.5	I	S
ATP synthase gamma chain	Q55SW7	ATP3/ Gamma subunit of the F1 sector of mitochondrial F1F0 ATP synthase	4.40E-62	S	+2	S
ATP synthase subunit beta	Q5KFU0	ATP2/ Beta subunit of the F1 sector of mitochondrial F1F0 ATP synthase	3.10E-203	-2	-1.5	+1.2
ATP synthase subunit alpha	Q5KFB9	ATP1/ Alpha subunit of the F1 sector of mitochondrial F1F0 ATP synthase	5.50E-197	-2	-1.7	+1.6
Chimeric spermidine synthase/saccharopine dehydrogenase	Q6RXX2	SPE3/ Spermidine synthase	2.60E-96	S	S	-2.5
Complex 1 protein	Q5KNR5	YGL006W-A/ Unknown	0.95	+2	--	S
Cytochrome c oxidase subunit 2	Q85SZ4	COX2/ Subunit II of cytochrome c oxidase	2.20E-76	I	I	+3
Electron carrier	Q5KNC7	CYC7/ Cytochrome c isoform 2	1.90E-38	+3*	+2.4	S
Importin beta-4 subunit	Q5KFR0	KAP123/ Karyopherin beta	4.00E-71	S	-2*	-1.6
Inorganic phosphate transporter	Q5K756	MIR1/ Mitochondrial phosphate carrier	8.10E-93	N	+2	+1.4
NADH dehydrogenase	Q5KN57	NDE1/ Mitochondrial external NADH	3.00E-118	--	--	-2

		dehydrogenase				
Pre-mRNA splicing factor	Q5KLG7	SMD3/ Core Sm protein Sm D3	3.00E-24	+2*	I	S
Structural molecule	Q5KLP2	ATP7/ Subunit d of the stator stalk of mitochondrial F1F0 ATP synthase	1.10E-38	-1.5	--	-2
Peripheral-type benzodiazepine receptor	Q5K6Y4	YLR154W-A/ Dubious open reading frame	0.27	+1.6	+2.6	+2
Ubiquinol-cytochrome C reductase complex core protein 2	Q5K8U4	QCR2/ Subunit 2 of the ubiquinol cytochrome-c reductase complex	1.40E-32	S	--	+4*
Voltage-dependent ion-selective channel	Q5KJP2	POR1/ Mitochondrial porin (voltage-dependent anion channel)	5.90E-42	+1.8	+2	N

[1] Obtained from Uniprot (http://www.uniprot.org/)

[2] Obtained from *Saccharomyces* Genome Database (http://www.yeastgenome.org/) or Clusters of Orthologous Groups of proteins (http://www.ncbi.nlm.nih.gov/COG/)

[3] Proteins with an E value of $\geq 10^{-9}$ were not included in the network analysis

[4] Based on normalised spectrum counts (ratio of FLC treated sample versus the untreated). “I” indicates induced proteins that present in treated samples only, “S” indicates suppressed proteins present in untreated controls only, “--“ indicates proteins that were absent in both treated and untreated samples, and “N” represents proteins with unchanged abundance level. Fold change with * indicates significant difference ($p < 0.05$).
